# Randomized phase III trial of intravenous and intraperitoneal paclitaxel with S-1 versus gemcitabine plus nab-paclitaxel for pancreatic ductal adenocarcinoma with peritoneal metastasis (SP study)

**DOI:** 10.1186/s13063-022-06049-7

**Published:** 2022-02-05

**Authors:** Tomohisa Yamamoto, Tsutomu Fujii, Satoshi Hirano, Fuyuhiko Motoi, Goro Honda, Kenichiro Uemura, Joji Kitayama, Michiaki Unno, Yasuhiro Kodera, Hiroki Yamaue, Toshio Shimokawa, Daisuke Hashimoto, So Yamaki, Hideyuki Yoshitomi, Fumihiko Miura, Hideki Ueno, Mitsugu Sekimoto, Sohei Satoi, on behalf of the Study Group of Pancreatic Ductal Adenocarcinoma with Peritoneal Metastasis

**Affiliations:** 1 Department of Surgery, Kansai Medical University, 2-3-1, Shin-machi, Hirakata, Osaka, 573-1191 Japan; 2 Department of Surgery and Science, Faculty of Medicine, Academic Assembly, University of Toyama, Toyama, Japan; 3 Department of Gastroenterological Surgery II, Hokkaido University Faculty of Medicine, Sapporo, Japan; 4 Department of Surgery I, Yamagata University, Yamagata, Japan; 5 Department of Surgery, Institute of Gastroenterology, Tokyo Women’s Medical University, Tokyo, Japan; 6 Department of Surgery, Graduate School of Biomedical and Health Sciences, Hiroshima University, Hiroshima, Japan; 7 Department of Surgery, Division of Gastroenterological, General and Transplant Surgery, Jichi Medical University, Tochigi, Japan; 8 Department of Surgery, Tohoku University Graduate School of Medicine, Sendai, Japan; 9 Gastroenterological Surgery, Nagoya University Graduate School of Medicine, Nagoya, Japan; 10 Second Department of Surgery, Wakayama Medical University, School of Medicine, Wakayama, Japan; 11 Clinical Study Support Center, Wakayama Medical University, School of Medicine, Wakayama, Japan; 12 Department of General Surgery, Graduate School of Medicine, Chiba University, Chiba, Japan; 13 Department of Surgery, Teikyo University Hospital, Mizonokuchi, Kawasaki, Japan; 14 Hepatobiliary and Pancreatic Oncology Division, National Cancer Center Hospital, Tokyo, Japan

**Keywords:** Pancreatic ductal carcinoma, Peritoneal metastasis, Peritoneal desemination, Intraperitoneal Paclitaxel, Randomized phase3 trial

## Abstract

The prognosis of pancreatic ductal carcinoma (PDAC) with peritoneal metastasis remains dismal. Systemic chemotherapy alone may not be effective, and the combination of intraperitoneal chemotherapy with systemic chemotherapy is expected to prolong the overall survival in patients with peritoneal metastasis. We have designed a randomized phase III trial to confirm the superiority of intravenous (i.v.) and intraperitoneal (i.p.) paclitaxel (PTX) with S-1 relative to gemcitabine plus nab-PTX (GnP), which is the current standard therapy for patients with metastatic PDAC. A total of 180 patients will be accrued from 30 institutions within 3 years. Patients will be randomly assigned in a 1:1 ratio to receive either i.v. and i.p. PTX with S-1 or GnP (target of 90 patients per group). The primary endpoint is overall survival; secondary endpoints are progression-free survival, response rate, proportion with negative peritoneal washing cytology during chemotherapy, proportion requiring conversion surgery, and adverse event profiles. Japan Registry of Clinical Trials jRCTs051180199 (https://jrct.niph.go.jp/).

## Introduction

Pancreatic ductal adenocarcinoma (PDAC) is the fourth leading cause of death in Japan, with a 5-year survival rate of less than 10% [1]. Surgical resection is the only curative treatment for pancreatic cancer, but the proportion of patients eligible for curative resection is only 25% [2, 3]. The majority (70–80%) of PDAC cases are locally advanced and metastatic diseases categorized as unresectable, which results in a limited prognosis.

The standard first-line therapies for patients with unresectable PDAC are gemcitabine plus nanoparticle albumin-bound (nab)-paclitaxel (PTX) [4] or FOLFIRINOX (folinic acid, 5-fluorouracil, irinotecan, and oxaliplatin) [5]. The median survival time (MST) and objective response rate (ORR) are 8.5 months and 23%, respectively, with gemcitabine plus nab-paclitaxel (GnP) [4] and 11.1 months and 32%, respectively, with FOLFIRINOX in patients with metastatic PDAC [5].

Peritoneal metastasis is defined as the presence of microscopic peritoneal dissemination (positive peritoneal washing cytology) and/or macroscopic peritoneal dissemination. Patients with peritoneal metastasis are generally treated with systemic chemotherapy with the same regimens as patients with other distant metastases. However, the presence of peritoneal metastasis is associated with the development of intestinal obstruction and massive ascites, which deprives patients of the opportunity to receive chemotherapy [6]. Consequently, these patients have a dismal prognosis of 4 to 10 weeks [6, 7]. Therefore, the development of a new effective treatment strategy is urgently needed. For the treatment of peritoneal dissemination, intraperitoneal (i.p.) chemotherapy seems to be advantageous compared with systemic chemotherapy due to a high drug concentration in the peritoneal cavity that can contact tumor nodules directly [8–11]. Kamei et al. demonstrated that i.p. administration of PTX nanoparticles in mice resulted in high accumulation in disseminated nodules, presumably due to superior penetrating activity directly into malignant tissue [12]. Ishigami et al. conducted a phase II study of weekly intravenous (i.v.) and i.p. PTX with S-1 in gastric cancer with peritoneal metastases, with remarkable results, such as an ORR of 56%, disappearance or marked decrease in malignant ascites in 62% of patients, and a 1-year overall survival (OS) rate of 78% [9]. Based on these results, we conducted a phase II multicenter trial to evaluate the clinical efficacy and tolerability of i.v. and i.p. PTX combined with S-1 in PDAC patients with peritoneal metastasis without other distant organ metastases. This trial regimen has achieved a promising response rate (RR) of 36% and a disease control rate (DCR) of 82% with acceptable toxicities, and the MST and 1-year OS rate were 16.3 months and 62%, respectively [13]. Moreover, conversion surgery for patients whose peritoneal metastasis disappeared was performed for 24.2% of the enrolled patients, and the MST in patients who received conversion surgery reached 27.8 months.

Based on the results of our phase II study, we designed a phase III trial to evaluate the superiority of i.v. and i.p. PTX combined with S-1 (S1-PTX) compared with GnP as a standard treatment in patients with PDAC with peritoneal metastasis.

## Protocol digest of study (SP study)

### Objectives

The primary objective of this study is to confirm the superiority of S1-PTX compared with GnP in patients with PDAC with peritoneal metastasis.

### Study design

The study is a multicenter, two-arm, open-label, randomized phase III trial.

### Endpoints

The primary endpoint is OS in all randomized patients. OS is calculated from the day of randomization to the day of death from any cause and censored at the last day that the patient is alive. The secondary endpoints are ORR, progression-free survival (PFS), proportion of patients with negative peritoneal washing cytology, alleviated cancer symptoms (intestinal ileus, ascites, hydronephrosis, etc.), decreased tumor marker levels, proportion of patients eligible for conversion surgery, % planned dose of chemotherapy, and safety (adverse event profile, etc.). PFS is defined as survival from the day of randomization to disease progression or death from any cause and is censored at the last day the patient is alive without any evidence of progression. Conversion surgery is planned for patients who are expected to have margin-negative resection with negative peritoneal washing cytology and disappearance of peritoneal dissemination during chemotherapy.

### Inclusion criteria

The following are the inclusion criteria:

1. PDAC by histological or cytological diagnosis
2. Presence of microscopic peritoneal metastasis during staging laparoscopy in patients with radiographically defined unresectable locally advanced PDAC or presence of macroscopic peritoneal dissemination on staging laparoscopy or open laparotomy in all types of PDAC
3. Chemo(radio)therapy-naive or within 3 months from initiation of chemo(radio)therapy and no progressive disease during the 3 months
4. Eastern Cooperative Oncology Group (ECOG) performance status of 0 or 1
5. Spared organ function satisfying the following laboratory data:
  - White blood cell count ≥ 3500/mm^3^ and < 12000/mm^3^
  - Neutrophils ≥ 2000/mm^3^
  - Hemoglobin ≥ 8.0 g/dl
  - Platelet count ≥ 100,000/mm^3^
  - Serum total bilirubin ≤ 2.0 mg/dl (or ≤ 3.0 mg/dl in patients with biliary drainage)
  - Aspartate aminotransferase (AST) ≤ 150 IU/l
  - Alanine aminotransferase (ALT) ≤ 150 IU/l
  - Creatinine ≤ 1.2 mg/dl
  - Creatinine clearance ≥ 50 ml/min
6. Adequate oral intake
7. Age 20–79 years
8. Provision of written, informed consent

### Exclusion criteria

The following are the exclusion criteria:

- Presence of metastasis in other distant organs, such as the liver, lungs, bone, or others, excluding the ovaries.
- Presence of microscopic peritoneal metastasis in patients with resectable or borderline resectable PDAC without macroscopic peritoneal dissemination.
- History of malignancy in the last 5 years. Patients with other malignancies are eligible if they were cured by surgery alone or surgery plus chemo(radio)therapy and have been continuously disease-free for at least 5 years.
- Allergy to chemotherapeutic agents (S-1, PTX, gemcitabine, nab-PTX).
- Unstable angina pectoris or myocardial infarction.
- Serious co-existing illness (ileus, pulmonary fibrosis, interstitial pneumonia, unstable diabetes mellitus, renal failure, liver cirrhosis, etc.).
- Massive ascites extending continuously from the pelvic cavity to the upper abdominal cavity.
- Bleeding in the alimentary tract with repetitive blood transfusion.
- Severe diarrhea.
- Psychiatric disease.
- Synchronous malignancy except for carcinoma in situ or intramucosal tumor after adequate curative treatment.
- Pregnancy, breast-feeding, or desire of a woman to preserve fertility.
- Regular use of flucytosine (increased risk of adverse events with a combined use of paclitaxel), phenytoin (increased risk of elevated phenytoin level and neurotoxic effect with a combined use of S-1), or warfarin (increased risk of bleeding events due to prolonged prothrombin time with a combined use of S-1).
- Cancer invasion to gastric or intestinal mucosa in the alimentary tract.

### Randomization

**Fig. 1:**
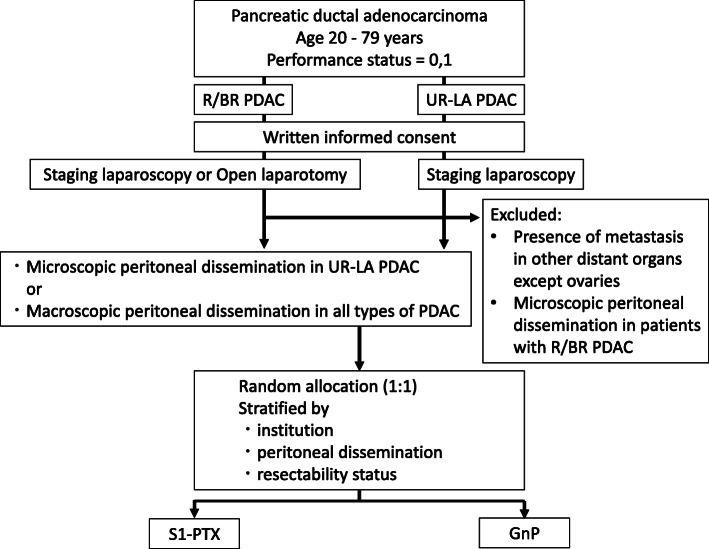
Schematic flowchart of the trial. PDAC, pancreatic ductal adenocarcinoma; S1-PTX, intravenous and intraperitoneal paclitaxel with S-1; GnP, gemcitabine plus nanoparticle albumin-bound (nab)-paclitaxel

After confirmation of eligibility, including written informed consent, eligible patients are registered centrally and assigned randomly to treatment. Central randomization and registration are carried out with an electronic data capture (EDC) system. After being assessed for eligibility at registration, patients are randomized centrally to either the S1-PTX arm or the GnP arm (Fig. 1). Randomization is performed by minimization methods to which the investigators are masked. Patients are stratified according to institution, macroscopic peritoneal dissemination, and resectability status of the primary tumor defined according to the NCCN guideline [14].

### Treatment

In the S1-PTX arm, PTX is administered i.v. at a dose of 50 mg/m^2^ and i.p. at 20 mg/m^2^ on days 1 and 8, and S-1 is administered orally at a dose according to body surface area (BSA), as follows: BSA < 1.25 m^2^, 40 mg; BSA 1.25–1.50 m^2^, 50 mg; and BSA > 1.50 m^2^, 60 mg, twice daily on days 1–14 of a 21-day cycle. PTX is diluted in 500 ml of normal saline and administered after administration of 500 ml of normal saline through the implanted peritoneal access port over 1 h concurrently with i.v. infusion after standard premedication.

Chemotherapy is started when patients’ recovery status fulfills the following criteria on day 1 of each cycle of treatment: neutrophil count > 1500/mm^3^, platelet count > 75,000/mm^3^, creatinine < 1.2 mg/dl, no febrile neutropenia, grade 1 or lower oral mucositis, diarrhea, and skin rash.

In the GnP arm, nab-PTX 125 mg/m^2^ is administered in combination with gemcitabine 1000 mg/m^2^ weekly for 3 weeks followed by 1 week of rest (4-week cycles).

Chemotherapy is started when patients’ recovery status fulfills the following criteria on day 1 of each cycle of treatment: neutrophil count > 1500/mm^3^, platelet count > 75,000/mm^3^, creatinine < 1.2 mg/dl, no febrile neutropenia, grade 1 or lower oral mucositis, diarrhea, and peripheral sensory neuropathy.

Two levels of dose modifications are permitted according to the criteria. If toxicity requiring dose modification occurs following the second dose reduction of either study drug, further treatment should be discontinued.

In both arms, protocol treatment will be continued until disease progression, unacceptable toxicity, conversion surgery with exceptional response, or patient refusal.

### Discontinuation criteria

The following are the discontinuation criteria:

- Patient withdrawal of consent for participation
- Adverse events directly attributable to the investigating treatment prohibiting resumption of treatment within 28 days
- Disease progression confirmed by diagnostic imaging, etc.
- Requirement for and initiation of radiation therapy
- Indication for and performance of surgery
- Discontinuation of the entire study

### Follow-up

Enhanced abdominal CT and chest CT for assessing tumor response according to RECIST ver.1.1 [15] and assessments of tumor markers (CA19-9, CA125, and CEA) are carried out every 8 to 9 weeks during protocol treatment in all randomized patients. In the S1-PTX arm, peritoneal (washing) cytology is evaluated every 8 to 9 weeks.

Physical examinations, complete blood counts (CBCs), and blood chemistry assessments are performed at each administration of i.v. chemotherapy in both groups. All adverse events are assessed according to the Common Terminology Criteria for Adverse Events, version 4.0. Quality of life is assessed with the EuroQOL-5 Dimension Questionnaire (EQ-5D) [16] and the European Organization for Research and Treatment of Cancer QLQ-C30 (EORTC QLQ-C30) [17] at baseline and every 8 to 9 weeks.

Data are collected via a case report form using an EDC system and paper and stored and managed securely by the data monitoring committee. To promote data quality, missing data will be pursued until received or confirmed as not available or until the trial reaches analysis.

### Study design and statistical analysis

This randomized trial is designed to confirm the superiority of S1-PTX in patients with PDAC with peritoneal metastasis. We assumed that the MST of GnP was 9.0 months and that of S1-PTX was 14.0 months based on previous studies [4, 13]. According to these assumed values, the hazard ratio was calculated to be 0.643. Given the assumption of a power of 80% or higher and a two-sided significance level of 0.05, the required minimum sample size was 85 patients per group. The planned accrual period is 3 years and the follow-up period is 1.5 years for the primary analysis. Accordingly, the sample size was set as 90 patients or more per group with an assumption that a few patients would become ineligible for this trial.

The survival analysis is based on the intent-to-treat population, which includes all eligible patients enrolled in this trial, with survival estimates calculated using the Kaplan-Meier method and compared using the stratified log-rank test. Survival estimates are presented with 95% confidence intervals. Hazard ratios and 95% confidence intervals will be estimated by the Cox proportional hazard model. *p* <0.05 will be considered significant.

The data monitoring committee and study coordinator (Clinical Study Support Center, Wakayama Medical University School of Medicine) will conduct central monitoring and will issue a monitoring report every 6 months to evaluate the study progress and improve data integrity and patient safety.

### Ethics

This study is performed in accordance with the Declaration of Helsinki. This protocol was approved by the Wakayama Medical University Hospital Clinical Research Review Board on October 18, 2019. This study has been registered with the Japan Registry of Clinical Trials, and the registration number is jRCTs051180199 (https://jrct.niph.go.jp/).

## Discussion

The prognosis of patients with PDAC with peritoneal metastasis remains extremely poor [6, 7]. The MST of these patients has been reported to be 4–10 weeks. In addition, most patients with PDAC with peritoneal metastasis suffer from massive ascites, have poor performance status, and receive less opportunity for chemotherapy [18]. One of the reasons for the poor prognosis is that only a small fraction of the systemically administered agents is delivered to the peritoneum. Thus, patients with peritoneal metastasis require prevention of the development of ascites and improved OS. Therefore, the development of an effective regimen of chemotherapy is mandatory.

S-1 is an oral fluoropyrimidine derivative. In Japan, S-1 is frequently used as an adjuvant therapy after pancreatectomy in patients with PDAC [19] or as a secondary treatment for unresectable PDAC [20]. In the GEST study, the MST was 9.7 months in patients with unresectable PDAC who received S-1 monotherapy as first-line treatment [21], and noninferiority of S-1 to gemcitabine with respect to OS was demonstrated. Moreover, S-1 and PTX share two favorable characteristics for the treatment of peritoneal metastasis: a high efficacy against diffuse-type adenocarcinoma that can be disseminated easily and a high rate of transition into the peritoneal cavity [22–24]. Therefore, the combination chemotherapy with S-1 and i.v. PTX is expected to be effective for peritoneal metastasis.

Intraperitoneal chemotherapy seems to be a reasonable approach to treat peritoneal metastasis directly. However, there has been little evidence of the superiority of i.p. PTX for PDAC with peritoneal metastasis. Previously, it was shown that i.p. PTX provided favorable clinical benefits in patients with peritoneal metastasis of other cancers and even PDAC [8, 9, 15, 25, 26]. Most notably, Ishigami et al. conducted a phase III study of weekly i.v. and i.p. PTX plus S-1 compared with S-1 plus cisplatin in gastric cancer with peritoneal dissemination. This trial unfortunately failed to show statistical superiority of i.p. PTX plus systemic chemotherapy owing to a crucial imbalance in the high amount of ascites in the experimental group and the crossover use of i.p. therapy in the control group [27]. In the area of PDAC with peritoneal dissemination, several clinical studies investigating the role of i.p. chemotherapy have been conducted since 2016 in Japan [25]. In a retrospective study to evaluate the clinical efficacy of i.v. and i.p. PTX combined with S-1 in comparison with S-1 or gemcitabine-based chemo(radio)therapy in chemo-naive patients with PDAC with peritoneal metastasis, implementation of the i.p. PTX regimen was closely associated with the prevention of ascites and higher resectability (30% vs 7%, *p* = 0.032), resulting in the improvement of OS (MST of 20 vs 10 months, *p* = 0.004). Two phase II studies of S-1 + PTX [13] and GnP + i.p. PTX [26] revealed promising overall survival (16.3 and 14.5 months, respectively) with a high proportion of conversion surgery (24% and 17%, respectively) and acceptable toxicity in patients with peritoneal metastasis. These promising results encouraged us to proceed with this phase III trial, which was initiated in February 2020.

We expect that this trial will show the superiority of S1-PTX compared with GnP and that S1-PTX will become a new standard therapy in patients with PDAC with peritoneal metastasis.

### Participating institutions (from north to south)

The following are the participating institutions:

Sapporo Medical University

Hokkaido University

Hakodate Municipal Hospital

Hirosaki University

Tohoku University

Yamagata University

Jichi Medical University

Gunma University

Tokyo Medical and Dental University

Juntendo University

Tokyo Medical University

Toho University Ohashi Medical Center

Cancer Institute Hospital

Seikeikai New Tokyo Hospital

Yokohama City University

Shinshu University

University of Toyama

Nagoya University

Shiga University of Medical Science

Kansai Medical University

Kindai University

Osaka City University

Nara Medical University

Wakayama Medical University

Tokushima University

Ehime University

Hiroshima University

Shimane University

Kyushu University

Kagoshima University

#### Dissemination policy

The results of the SP study will be submitted to a peer-reviewed journal and will be presented at national and international conferences regardless of the trial outcomes.

#### Recruitment

To achieve adequate participant enrollment to reach the target sample size within the study period, 30 Japanese high-volume centers will participate in the SP study.

#### Trial status

Patient enrollment began on February 04, 2020. The study completion date is estimated to be July 2024.

## Data Availability

The datasets used and/or analyzed will be available from the corresponding author on reasonable request after the current study is complete.
